# Persistent Sexual and Psychological Symptoms After Finasteride Discontinuation: A Cross-Sectional Observational Study

**DOI:** 10.3390/jcm15082947

**Published:** 2026-04-13

**Authors:** Paweł Jędrzejczyk, Tomasz Ząbkowski, Jarosław Ratajski, Kamil Ciechan, Tomasz W. Kaminski, Patryk Uciechowski, Tomasz Syryło

**Affiliations:** 1 Gen Clinic, 44/U1 Wilhelma Roentgena Street, 02-781 Warsaw, Poland; 2 Department of General, Functional and Oncological Urology, Military Institute of Medicine—National Research Institute, 128 Szaserów Street, 04-141 Warsaw, Poland; 3 Department of Uro-Oncology and Minimally Invasive Urology, Bielanski Hospital Named After Father Jerzy Popiełuszko, 80 Cegłowska Street, 01-809 Warsaw, Poland; 4 Warsaw Bar Association, 15/16 Żytnia Street, 01-014 Warsaw, Poland; 5 Hemostasis and Thrombosis Program, Versiti Blood Research Institute, Milwaukee, WI 53226, USA; 6 Outpatient Clinics, Specialist Laboratories, Urology Outpatient Clinic, Military Institute of Medicine—National Research Institute, 128 Szaserów Street, 04-141 Warsaw, Poland

**Keywords:** finasteride, post-finasteride syndrome, sexual dysfunction

## Abstract

**Background:** Persistent sexual and psychological symptoms after finasteride discontinuation have been reported; however, factors associated with symptom severity remain insufficiently characterized. **Methods:** This cross-sectional study included 129 adult men with prior finasteride exposure for male pattern hair loss or benign prostatic hyperplasia. Sexual function, depressive symptoms, and anxiety were assessed using the International Index of Erectile Function (IIEF), Patient Health Questionnaire-9 (PHQ-9), and Generalized Anxiety Disorder-7 (GAD-7), respectively. Associations between clinical variables (age, treatment duration, cumulative exposure, and indication) and symptom severity were evaluated using univariate and multivariable regression analyses. **Results:** The median treatment duration was 24 months (IQR: 12.5–36), and the median time from discontinuation to evaluation was 8 months (IQR: 1–17). Erectile function remained stable over time (mean IIEF: 15.2 ± 0.46 at baseline vs. 15.4 ± 0.47 at 6 months). Depressive symptoms decreased from 12.4 ± 0.41 to 9.1 ± 0.41, and anxiety scores from 3.29 ± 0.23 to 2.54 ± 0.20 over the same period, without normalization in most patients. In multivariable analyses, higher cumulative exposure and older age were independently associated with lower IIEF scores and higher PHQ-9 and GAD-7 scores. **Conclusions:** In this symptomatic cohort, greater cumulative finasteride exposure and older age were associated with more severe sexual and psychological symptoms after treatment discontinuation. These findings highlight the need for prospective studies to better define risk factors and long-term outcomes.

## 1. Introduction

Finasteride is a selective inhibitor of 5-α-reductase (5-ARI), the enzyme that converts testosterone to dihydrotestosterone (DHT), a potent androgen active in androgen-dependent tissues [1,2]. By selectively inhibiting the type II isoenzyme, finasteride substantially reduces DHT concentrations in the prostate and hair follicles, whereas circulating testosterone levels generally remain within the physiological range [3].

This mechanism supports its two primary clinical indications. In benign prostatic hyperplasia (BPH), sustained DHT suppression reduces prostate volume, improves lower urinary tract symptoms, and lowers the risk of disease progression, particularly in patients with larger baseline prostate volumes [4,5,6]. In male pattern hair loss (MPHL), inhibition of follicular DHT activity limits hair follicle miniaturization and may stabilize disease progression during continuous therapy [7,8].

Despite proven efficacy, concerns regarding the safety profile of finasteride have increased. Sexual adverse effects were initially considered uncommon and reversible after discontinuation; however, accumulating clinical, observational, and pharmacovigilance evidence indicates that some patients develop persistent symptoms after treatment cessation. These manifestations include sexual dysfunction, neuropsychiatric symptoms, and nonspecific somatic complaints, collectively termed post-finasteride syndrome (PFS). Its nosological status remains debated because of heterogeneous definitions, lack of standardized diagnostic criteria, and limited prospective data [1,2,9,10].

Finasteride is prescribed to clinically distinct populations. Patients with BPH are typically older adults and often have comorbidities, whereas those treated for MPHL are generally younger males without significant underlying disease and may receive prolonged therapy for primarily cosmetic indications [2,6,11]. These population differences may influence both the risk and clinical expression of adverse effects and their persistence after discontinuation.

Particular concern surrounds finasteride use in MPHL, where long-term exposure frequently occurs during periods of full hypothalamic–pituitary–gonadal axis activity [1,2]. Chronic DHT suppression at this stage may predispose susceptible individuals to persistent alterations in androgen-dependent tissues and neuroendocrine regulation. Notably, persistent symptoms have been reported despite normalization of serum androgen levels, suggesting mechanisms beyond androgen deficiency alone [3].

Experimental and clinical data indicate that finasteride may disrupt neurosteroid metabolism and central nervous system signaling. Inhibition of 5-α-reductase alters synthesis of neuroactive steroids, including pregnenolone, progesterone, and dehydroepiandrosterone, which regulate sexual function, mood, and cognition. Disturbance of these pathways may contribute to the concurrent sexual and neuropsychiatric symptoms observed in some patients after discontinuation [2,3].

Treatment duration and cumulative exposure have also been proposed as determinants of post-treatment outcomes. Observational evidence suggests that longer exposure may increase the likelihood of persistent symptoms, although prospective confirmation is lacking [1,9]. Patient age and individual biological characteristics, including baseline and post-treatment hormonal parameters, may further shape clinical presentation—sexual, psychological, or somatic—supporting the concept of PFS as a heterogeneous rather than uniform entity [2,3,12].

Collectively, these findings underscore the need to systematically evaluate associations between finasteride exposure characteristics and the severity of sexual and psychological symptoms in patients reporting persistent symptoms after discontinuation. Use of standardized instruments to assess sexual function and mental health may enable objective characterization of symptom burden and improve understanding of the potential long-term effects of 5-α-reductase inhibitor therapy.

## 2. Materials and Methods

This observational, cross-sectional study combined retrospective assessment of finasteride exposure with prospective evaluation of persistent symptoms after discontinuation. The objective was to examine associations between exposure characteristics and the severity of persistent sexual, depressive, and anxiety symptoms.

A total of 129 male patients with prior finasteride use were included. Eligible participants had received finasteride at any dose for male pattern hair loss (MPHL) or benign prostatic hyperplasia (BPH) and had discontinued therapy before enrollment. Clinical assessment occurred after cessation to capture symptoms persisting beyond the acute withdrawal phase. In this study, ‘persistent symptoms’ were operationally defined as symptoms reported at the time of clinical evaluation following treatment discontinuation, rather than based on a predefined minimum duration. Information on the reasons for finasteride discontinuation (e.g., adverse effects, perceived lack of efficacy, or other factors) was not systematically collected and therefore was not included in the analysis.

Participants were stratified by therapeutic indication (MPHL vs. BPH) and age at exposure. Age was dichotomized at the cohort median (49 years) to ensure balanced subgroup comparisons.

Finasteride-related variables were collected retrospectively and included total treatment duration (months) and time from discontinuation to clinical evaluation. Cumulative exposure was calculated as the result of the reported daily finasteride dose and the duration of treatment (in months), providing an integrated measure of total drug exposure. Two temporal variables were analyzed independently: 1. duration of finasteride exposure, defined as total treatment length, and 2. time since discontinuation, defined as the interval between cessation and symptom assessment. These variables were evaluated separately to distinguish exposure-related effects from post-withdrawal dynamics.

Clinical evaluation focused on sexual and psychiatric outcomes using structured interviews based on validated instruments. Sexual function was assessed with the International Index of Erectile Function (IIEF). Depressive symptoms were measured using the Patient Health Questionnaire-9 (PHQ-9), and anxiety symptoms with the Generalized Anxiety Disorder-7 (GAD-7). Instruments were administered in a clinician-guided format to enhance consistency and interpretability. Patients also reported persistent somatic symptoms after discontinuation.

Endocrine assessment included total testosterone, dihydrotestosterone (DHT), luteinizing hormone (LH), follicle-stimulating hormone (FSH), sex hormone-binding globulin (SHBG), estradiol, and prolactin. Measurements were performed in a certified laboratory using standard assays. Hormonal parameters were analyzed in relation to sexual and psychiatric symptom severity to contextualize findings within an endocrine framework.

Symptom trajectory was explored by collecting data on symptom duration and perceived change over time. Severity was evaluated at 1, 3, and 6 months after discontinuation, allowing exploratory characterization of post-treatment patterns independent of prior exposure length.

### Statistical Analysis

Statistical analyses were conducted using GraphPad Prism (version 10, GraphPad Software, San Diego, CA, USA). Distribution of continuous variables was assessed with the Shapiro–Wilk test. Continuous data are presented as mean ± standard error of the mean (SEM) or median with interquartile range (IQR), as appropriate. Categorical variables are reported as counts and percentages.

Longitudinal comparisons of IIEF, PHQ-9, and GAD-7 scores across baseline, 1, 3, and 6 months were performed using repeated-measures analysis of variance (ANOVA) with Tukey post hoc testing. Although some variables were non-normally distributed, repeated-measures ANOVA was applied because of the sample size and approximately continuous scale behavior; this method is robust to moderate non-normality.

Between-group comparisons (e.g., stratified by age, cumulative exposure, testosterone concentration, or indication) used the unpaired Student’s *t*-test or Mann–Whitney U test for continuous variables, depending on distribution and variance. Categorical variables were analyzed using the chi-square or Fisher’s exact test, as appropriate.

For subgroup analyses, the cohort was dichotomized at median values of age, cumulative exposure, and serum testosterone to ensure balanced groups and statistical stability. Associations among clinical, hormonal, lifestyle, and patient-reported variables were examined using Spearman’s rank correlation coefficients, with strength interpreted according to established thresholds and visualized via correlation heatmaps.

Independent predictors of persistent symptom severity were identified using univariate and multivariable logistic regression. Variables with clinical relevance or *p* < 0.10 in univariate analysis were entered into multivariable models, including age, exposure duration, cumulative dose, indication, hormonal parameters, and selected lifestyle factors. Results are reported as odds ratios (ORs) with 95% confidence intervals (CIs). All tests were two-sided, and *p* < 0.05 was considered statistically significant. Given the observational design and use of anonymized clinical data, formal ethics committee approval was not required. All participants provided informed consent for research use of their data.

## 3. Results

The analysis included 129 male patients with prior finasteride use who reported persistent symptoms after discontinuation, representing a preselected symptomatic cohort. The mean age at clinical evaluation was 47.1 ± 1.46 years; median age was 49 years (IQR: 31.5–62).

Median treatment duration was 24 months (IQR: 12.5–36). The median interval between discontinuation and assessment was 8 months (IQR: 1–17), indicating that symptoms were present well beyond the immediate post-discontinuation period in most patients. Cumulative exposure, calculated as daily dose and treatment duration, showed marked interindividual variability (median: 44 units; IQR: 18.5–153).

In 64% of patients, symptoms began during therapy or immediately after discontinuation. Sleep disturbances were reported by 58% of participants, and chronic fatigue by 67%; however, these findings reflect symptom frequency within a preselected symptomatic cohort. Mean body mass index (BMI) was 26.1 ± 0.28 kg/m^2^. Current smoking was reported by 21% of patients, and regular alcohol consumption by 66%. Baseline cohort characteristics are summarized in Table 1.

Follow-up assessment of sexual function, depressive symptoms, and anxiety demonstrated partial improvement; however, full normalization was not achieved in most patients.

**Figure 1:**
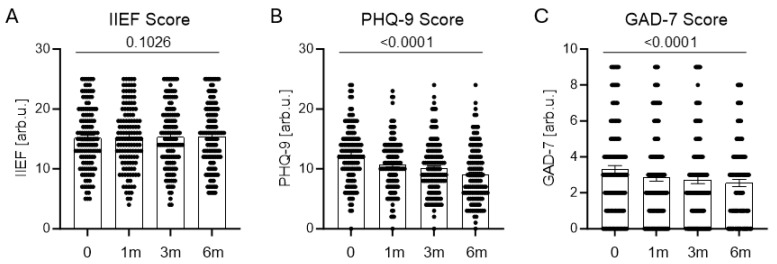
Longitudinal changes in sexual function, depressive symptoms, and anxiety after finasteride discontinuation. (**A**) International Index of Erectile Function (IIEF), (**B**) Patient Health Questionnaire-9 (PHQ-9), and (**C**) Generalized Anxiety Disorder-7 (GAD-7) scores measured at baseline (0), 1 month (1 m), 3 months (3 m), and 6 months (6 m). Each dot represents an individual patient; bars indicate mean ± SEM. *p* values were calculated using repeated-measures ANOVA with Tukey’s post hoc test.

Erectile function, assessed using the International Index of Erectile Function (IIEF), remained stable throughout follow-up, indicating persistent sexual dysfunction. Mean IIEF scores were 15.2 ± 0.46 at baseline, 15.3 ± 0.47 at 1 month, 15.3 ± 0.47 at 3 months, and 15.4 ± 0.47 at 6 months after finasteride discontinuation (Figure 1A).

Depressive symptom severity, measured using the Patient Health Questionnaire-9 (PHQ-9), decreased progressively from a mean baseline score of 12.4 ± 0.41 to 10.7 ± 0.38 at 1 month, 10.1 ± 0.39 at 3 months, and 9.1 ± 0.41 at 6 months. Despite this decline, mean scores remained within the moderate depressive symptom range throughout the observation period (Figure 1B).

Anxiety symptoms, assessed using the Generalized Anxiety Disorder-7 (GAD-7), showed a similar trend. Mean scores decreased from 3.29 ± 0.23 at baseline to 2.54 ± 0.20 at 6 months, indicating modest improvement without complete symptom resolution (Figure 1C).

Subjective improvement was greatest during the first month after treatment discontinuation. Thereafter, symptom trajectories stabilized, and most patients reported no further meaningful improvement.

Mean total testosterone was 18.9 ± 0.46 nmol/L, and mean dihydrotestosterone (DHT) was 39.6 ± 1.4 ng/dL. These values provided objective endocrine context for persistent symptoms.

Univariate analyses showed that testosterone levels did not significantly differentiate sexual, depressive, or anxiety symptom severity and were not associated with smoking or alcohol consumption.

**Figure 2:**
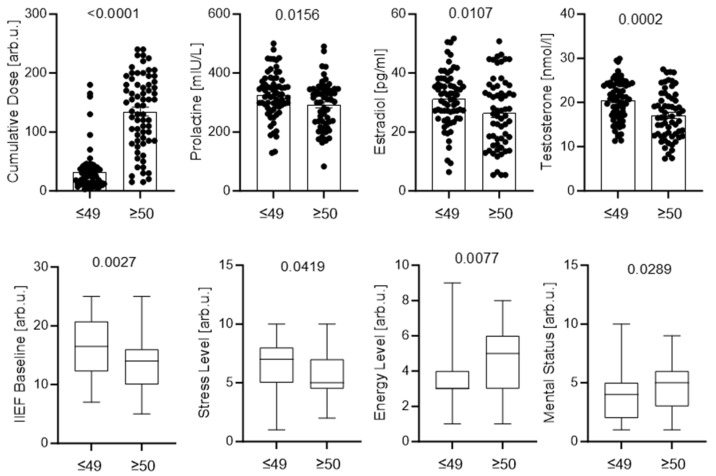
Clinical, hormonal, and patient-reported parameters stratified by age. Patients were divided into ≤49 and ≥50 years groups. Panels show cumulative finasteride exposure, prolactin, estradiol, testosterone, baseline IIEF, stress level, energy level, and mental status. Individual data points with mean ± SEM (**top panels**) or box plots (**bottom panels**) are presented. *p* values were calculated using appropriate parametric or nonparametric tests.

Comparative analyses identified significant phenotypic differences between patients aged <50 years and ≥50 years. Smoking prevalence was higher among patients aged ≥50 years (*p* = 0.009), whereas alcohol consumption did not differ significantly between age groups. Additional comparisons between these subpopulations are presented in Figure 2.

**Figure 3:**
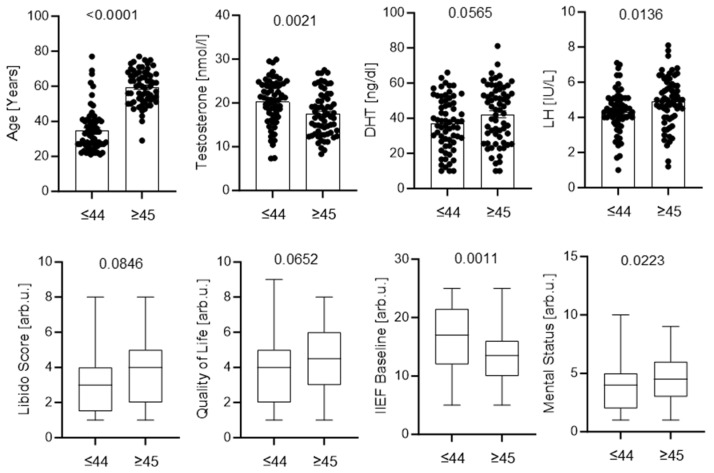
Demographic, hormonal, and clinical parameters stratified by testosterone levels. Patients were divided into low and high testosterone groups based on the cohort median. Panels show age, DHT, LH, prolactin, cumulative finasteride exposure, BMI, baseline IIEF score, and stress level. Individual data points with mean ± SEM (**top panels**) or box plots (**bottom panels**) are presented. *p* values were calculated using appropriate parametric or nonparametric tests.

The cohort was also stratified by median testosterone levels to assess hormonal and clinical differences. Patients with higher testosterone were significantly younger and had higher DHT levels and better baseline erectile function, whereas cumulative drug exposure was lower. No significant differences were observed in LH, prolactin, BMI, or perceived stress; however, trends toward lower LH and BMI were observed in the high-testosterone subgroup (Figure 3).

**Figure 4:**
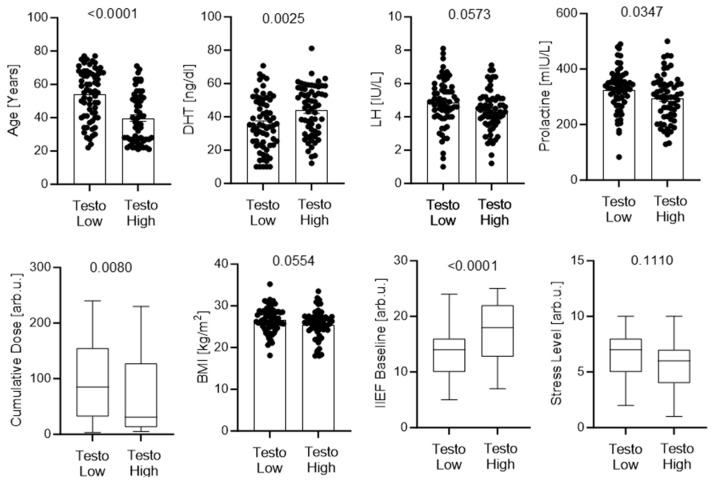
Demographic, hormonal, and patient-reported parameters stratified by cumulative finasteride exposure. Patients were divided into low and high cumulative exposure groups based on the cohort median. Panels show age, testosterone, DHT, LH, libido score, quality of life, baseline IIEF score, and mental status. Individual data points with mean ± SEM (**top panels**) or box plots (**bottom panels**) are presented. *p* values were calculated using appropriate parametric or nonparametric tests.

The cohort was further stratified by median cumulative dose to evaluate dose–response relationships. Patients with higher cumulative exposure were significantly older and had lower testosterone, higher LH, and poorer baseline erectile function, along with worse mental status scores. Trends toward lower libido and quality of life were also observed in this group. DHT levels showed a borderline increase, suggesting adaptive changes in androgen metabolism (Figure 4).

No significant differences in smoking or alcohol use were observed when stratifying patients by cumulative finasteride exposure or testosterone levels. Testosterone concentration did not significantly differentiate health-related behaviors or symptom severity in sexual or psychiatric domains.

Analysis of lifestyle factors showed limited influence on primary outcomes. Smoking was primarily associated with age and was not significantly correlated with testosterone, cumulative finasteride dose, or symptom severity measured by IIEF, PHQ-9, and GAD-7. Alcohol consumption was not significantly associated with age, hormonal parameters, or cumulative drug exposure.

**Figure 5:**
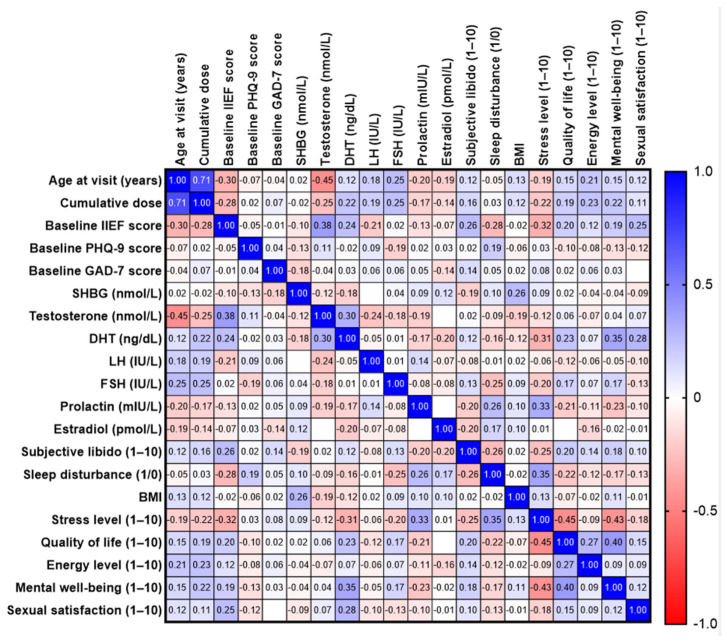
Spearman’s correlation matrix of demographic, hormonal, and patient-reported variables. Heatmap showing Spearman’s correlation coefficients (ρ) between clinical, hormonal, and questionnaire-derived parameters. Color intensity represents the strength and direction of associations (blue: positive, red: negative).

Spearman’s rank correlation analysis across the cohort identified several significant relationships. Age was strongly positively correlated with cumulative dose and moderately negatively correlated with testosterone. Baseline erectile function was positively associated with testosterone and DHT. Higher perceived stress was strongly associated with poorer quality of life and mental well-being. Prolactin showed a moderate positive correlation with stress and sleep disturbance. It should be noted that symptoms such as sleep disturbance and fatigue are nonspecific and may be prevalent in the general population, particularly in older individuals. Sexual satisfaction was positively correlated with testosterone and baseline IIEF, supporting the clinical relevance of androgen status in symptom severity (Figure 5).

Regression analyses identified finasteride exposure variables, particularly treatment duration and cumulative dose, along with patient age, as the strongest predictors of symptom severity. In contrast, lifestyle factors contributed minimally to the persistence and severity of sexual and psychiatric symptoms.

## 4. Discussion

This study characterizes a cohort of patients reporting persistent sexual and psychological symptoms after finasteride discontinuation and identifies cumulative drug exposure and age as key factors associated with symptom severity. The most consistent finding was the association between longer cumulative exposure and poorer erectile function, measured by IIEF, as well as greater depressive (PHQ-9) and anxiety (GAD-7) symptom severity. These relationships were especially evident in regression analyses of the entire cohort, indicating that exposure-related factors are central determinants of persistent symptoms.

An important consideration is the heterogeneity of the study population, particularly with respect to age and treatment indication. Finasteride is prescribed to clinically distinct groups, including younger men treated for male pattern hair loss and older men treated for benign prostatic hyperplasia. These populations may differ in baseline sexual function, hormonal milieu, psychological context, and perception of symptoms. In the present cohort, the median age was 49 years, and younger individuals, particularly those under 30 years of age, were underrepresented. Therefore, the findings may not fully reflect the clinical characteristics or symptom burden observed in younger populations, in whom concerns related to sexual function and quality of life may differ. Retaining both MPHL and BPH patients was intentional to reflect real-world clinical use of finasteride. Excluding specific subgroups, such as younger patients, would introduce additional selection bias and further limit the generalizability of the findings.

Longitudinal analysis showed modest improvement in depressive and anxiety symptoms at 1, 3, and 6 months after discontinuation; however, improvement was gradual and incomplete in many patients. Erectile function remained largely unchanged, suggesting persistent rather than transient dysfunction in a subset of patients. This finding supports the hypothesis that post-finasteride symptoms extend beyond short-term withdrawal effects.

Age-stratified analyses revealed additional clinically relevant differences, supporting the concept that post-finasteride symptoms may manifest differently across age groups. However, these findings should be interpreted cautiously given the limited representation of younger patients in the cohort. Patients aged ≥50 years more frequently exhibited unfavorable lifestyle factors, particularly cigarette smoking. Although these factors may influence clinical presentation, they did not fully explain differences in sexual or psychological symptom severity, indicating that age-related vulnerability reflects additional biological or clinical determinants.

Circulating testosterone levels were not significantly associated with smoking, alcohol consumption, or symptom severity. Comparisons between patients with lower and higher testosterone also showed no significant differences in sexual or psychological outcomes. These findings suggest that persistent symptoms cannot be explained solely by androgen deficiency and may involve more complex neuroendocrine, central nervous system, or psychosexual mechanisms.

Clinically, these results identify cumulative finasteride exposure and patient age as key modifiers of persistent symptom risk. The lack of normalization in IIEF, PHQ-9, and GAD-7 scores within six months suggests that symptoms may follow a chronic course in some patients rather than resolving spontaneously.

These findings have important implications for clinical decision-making, particularly in younger male patients treated for male pattern hair loss, where therapeutic benefit is primarily cosmetic. The results support comprehensive patient counseling before treatment initiation, including discussion of potential long-term sexual and psychological adverse effects. Monitoring should include systematic evaluation of mental health and sexual function during and after therapy.

Lifestyle factors such as smoking, particularly in older patients, may help guide individualized management and counseling. However, these factors alone do not explain symptom persistence, reinforcing the multifactorial nature of post-finasteride outcomes.

The limitations of this study should be acknowledged to provide appropriate context for interpretation of the findings. In particular, the relatively modest sample size, potential selection and recall biases inherent to the observational design, and limited availability of certain clinical and longitudinal data may influence the robustness and generalizability of the results. In particular, although the cohort size was comparable to those reported in similar studies of persistent post-finasteride symptoms, it may have limited statistical power for subgroup analyses and reduced the generalizability of the findings. Although the overall cohort included 129 participants, the relatively smaller number of younger patients treated for male pattern hair loss may have reduced the statistical power of indication-specific subgroup analyses and limited the generalizability of these comparisons. Importantly, the study cohort consisted exclusively of patients reporting persistent symptoms after finasteride discontinuation. As a result, the present design does not allow estimation of the prevalence, incidence, or relative risk of post-finasteride symptoms in the general population of treated individuals. This selection inherently introduces bias toward more severe or persistent cases and limits the generalizability of the findings. Accordingly, the results should be interpreted as describing associations within a symptomatic cohort rather than population-level risk. A further limitation is the lack of a standardized definition of “persistent” symptoms in the context of post-finasteride syndrome. In the present study, persistence was defined operationally based on symptom presence at the time of evaluation, and future studies should aim to establish consensus criteria, including minimum duration thresholds. An additional limitation is the lack of systematically collected data on baseline sexual function prior to finasteride initiation, as well as relevant comorbidities such as diabetes mellitus, hypertension, and concomitant medication use, all of which may influence sexual and psychological outcomes. The absence of these variables limits the ability to fully adjust for potential confounding factors. As a result, the observed associations between finasteride exposure and symptom severity should be interpreted with caution, as unmeasured confounding cannot be excluded. Younger patients, particularly those under 30 years of age, were underrepresented in this cohort, which may limit the applicability of the findings to populations most commonly treated for male pattern hair loss. Additionally, detailed information on the reasons for treatment discontinuation was not available. As discontinuation may have been influenced by adverse effects, patient perception, or other factors, this may introduce heterogeneity and potential bias in the study population.

## 5. Conclusions

This study highlights that sexual, psychological, and somatic symptoms may persist for months in a subset of patients reporting post-finasteride symptoms. Persistent erectile dysfunction and elevated depressive and anxiety symptoms, assessed using standardized scales, indicate that adverse effects may extend beyond active drug exposure.

Cumulative finasteride exposure was a key determinant of clinical outcomes. Higher cumulative dose was associated with poorer sexual function and greater depressive and anxiety severity, suggesting that total pharmacologic burden contributes to persistent symptoms. Patient age was an important modifying factor. Older adults more frequently reported persistent sexual symptoms and had a higher prevalence of unfavorable lifestyle factors, including smoking. However, persistent symptoms also occurred in younger patients, indicating risk across age groups. Longitudinal assessment showed gradual but incomplete improvement in depressive and anxiety symptoms, whereas recovery of sexual function was limited and heterogeneous. These findings highlight the variable and potentially chronic nature of post-finasteride symptom trajectories.

Clinically, these results emphasize the importance of careful risk–benefit assessment before initiating finasteride, considering patient age, expected treatment duration, and potential long-term effects. Monitoring of sexual and psychological symptoms should continue during and after treatment. Future research should prioritize prospective, long-term studies incorporating detailed endocrine, neurobiological, and psychosocial evaluation to better define risk factors, mechanisms, and recovery patterns associated with persistent finasteride-related symptoms.

## Figures and Tables

**Table 1:** Baseline demographic, clinical, hormonal, and patient-reported characteristics of the study population (*n* = 129). Continuous variables are presented as mean ± standard error of the mean (SEM), median, and interquartile range (25th–75th percentile). Categorical variables are reported as counts. IIEF, International Index of Erectile Function; PHQ-9, Patient Health Questionnaire-9; GAD-7, Generalized Anxiety Disorder-7; SHBG, sex hormone-binding globulin; DHT, dihydrotestosterone; LH, luteinizing hormone; FSH, follicle-stimulating hormone; BMI, body mass index.

Parameter (*n* = 129)	Mean	±SEM	Median	25%	75%
Age at visit (years)	47.1	1.46	49	31.5	62
Daily dose (1/5 mg/day)	59—1 mg/70—5 mg				
Continuous Treatment	100—Yes/29—No				
Treatment duration (months)	24.5	1.14	24	12.5	36
Cumulative dose (dose × months)	81.9	6.38	44	18.5	153
Time since discontinuation (months)	9.51	0.715	8	1	17
Symptom onset before/during therapy (0/1)	46—No/83—Yes				
Currently on medication (1/0)	100—0/29—1				
Baseline IIEF score	15.2	0.464	15	11	19
ED severity	1.9	0.117			
Baseline PHQ-9 score	12.4	0.411	12	10	15
Depression severity	3.08	0.0806	3	3	4
Baseline GAD-7 score	3.29	0.229	N/A	N/A	N/A
SHBG (nmol/L)	38.4	1.02	38.1	31.1	43.2
Testosterone (nmol/L)	18.9	0.461	19.1	14.8	23.8
DHT (ng/dL)	39.6	1.4	38.8	26.6	52.9
LH (IU/L)	4.61	0.118	4.6	3.95	5.45
FSH (IU/L)	4.71	0.152	4.5	3.5	5.65
Prolactin (mIU/L)	310	7.08	318	256	354
Estradiol (pmol/L)	106	3.59	107	80	132
Estradiol (pg/mL)	29	0.957	29.1	22.2	36
Subjective libido (1–10)	3.26	0.162	3	2	5
Sleep disturbance (1/0)	75—Yes/54—No				
Fatigue (1/0)	86—Yes/43—No				
BMI (kg/m^2^)	26.1	0.28	26.1	24.5	28.3
Physical activity (1/2/3)	69/39/21				
Physical activity before treatment (1/2/3)	37/54/38				
Current smoker (1/0)	27/102				
Alcohol consumption (1/0)	85/44				
Stress level (1–10)	6.05	0.176	6	5	7
Quality of life (1–10)	4.04	0.163	4	3	5
Energy level (1–10)	4.14	0.16	4	3	6
Mental well-being (1–10)	4.16	0.166	4	3	5.5
Sexual satisfaction (1–10)	3.28	0.136	3	2	4

## Data Availability

The raw data supporting the conclusions of this article will be made available by the authors on request.
